# Mussel-Inspired Carboxymethyl Chitosan Hydrogel Coating of Titanium Alloy with Antibacterial and Bioactive Properties

**DOI:** 10.3390/ma14226901

**Published:** 2021-11-15

**Authors:** Yanru Ren, Xiaoyan Qin, Mike Barbeck, Yi Hou, Haijun Xu, Luo Liu, Chaoyong Liu

**Affiliations:** 1 Beijing Bioprocess Key Laboratory, Beijing University of Chemical Technology, Beijing 100029, China; yanruren_sky@163.com (Y.R.); qxy19970425@163.com (X.Q.); houyi@iccas.ac.cn (Y.H.); hjxu@mail.buct.edu.cn (H.X.); 2 Institute of Material Science and Technology, Technical University of Berlin, Hardenbergstrasse 40, Sekr. BA3, 10623 Berlin, Germany; mike.barbeck@berlinanalytix.com

**Keywords:** carboxymethyl chitosan, gallic acid, titanium alloy, DMTMM, HRP/H_2_O_2_, catechol group, hydrogel coating, antibacterial

## Abstract

Infection-related titanium implant failure rates remain exceedingly high in the clinic. Functional surface coating represents a very promising strategy to improve the antibacterial and bioactive properties of titanium alloy implants. Here, we describe a novel bioactive surface coating that consists of a mussel-inspired carboxymethyl chitosan hydrogel loaded with silver nanoparticles (AgNPs) to enhance the bioactive properties of the titanium alloy. The preparation of hydrogel is based on gallic acid grafted carboxymethyl chitosan (CMCS-GA) catalyzed by DMTMM (4-(4,6-dimethoxy-1,3,5-triazin-2-yl)-4-methylmorpholinium chloride). To build a firm bonding between the hydrogel and titanium alloy plate, a polydopamine layer was introduced onto the surface of the titanium alloy. With HRP/H_2_O_2_ catalysis, CMCS-GA can simply form a firm gel layer on the titanium alloy plate through the catechol groups. The surface properties of titanium alloy were characterized by scanning electron microscope (SEM), X-ray photoelectron spectroscopy (XPS), and water contact angle. Silver nanoparticles were loaded into the gel layer by in situ reduction to enhance the antibacterial properties. In vitro antibacterial and cell viability experiments showed that the AgNPs-loaded Ti-gel possesses excellent antibacterial properties and did not affect the proliferation of rabbit mesenchymal stem cells (MSCs).

## 1. Introduction

Titanium and its alloys have become much more popular than other metals in clinics [1], for example, in orthopedic and cardiovascular implants, owing to their superior tissue compatibility, mechanical properties, and corrosion resistance [2]. Despite clean surgical procedures being followed and modern antibiotic regimes being used, the infection-related titanium implant failure rates remain exceedingly high in the clinic. For example, they account for approximately 14% of total implant failures in dental implant therapy. Many studies have confirmed that the formation of bacterial biofilm on the surface of titanium implants can cause serious inflammation and cause the loss of osseointegration, which will eventually cause the implant to loosen and fall off. Therefore, building an antibacterial coating on a titanium substrate is an effective strategy to prevent implant infection and implant failure, and also avoid the side effects of systemic administration on organs.

To date, various strategies have been reported to modify titanium surfaces with antibacterial properties, which mainly include: (1) loading of antibacterial drugs, such as antibiotics [3], or attaching antimicrobial peptides [4] to the surface; (2) introducing inorganic antibacterial metal elements such as F, Cu, Zn, or Ag by alloy or modification [5]; (3) applying a coating of antibacterial polymers [6]. Antibacterial drugs such as antibiotics are generally combined with the substrate through covalent bonding [7] or non-covalent adsorption [8,9,10]. However, these methods are not only prone to drug resistance, but also usually have a burst phase at the initial stage of drug release, which prevents long-term antibacterial effects. Many methods have also been performed to introduce inorganic antibacterial metal elements to titanium surfaces, such as plasma spraying [11], micro-arc oxidation [12], and ultrasonic spray coating [3]. Compared with organic antibacterial drugs, metal elements exhibit broad-spectrum antibacterial activity, long acting time, and no drug resistance. But these methods always lead to sacrificed mechanical property and biocompatibility [13]. Antibacterial polymer is a type of high molecular antibacterial agent, represented by chitosan. Layer-by-layer self-assembly (LBL) [14] and the sol-gel method [15] are common methods for preparing polymer coatings. Hydrogels not only possess viscoelasticity, low toxicity, and biocompatibility [16,17], but their unique swelling properties are conducive to sustained drug release. Thus, using them is an effective strategy to form a composite antibacterial coating. In particular, it has been proven that the in situ hybridization of AgNPs in the hydrogel matrix can reduce the unwanted agglomeration and burst leakage [5,18,19]. One key point for hydrogel application is to form robust bonding between hydrogels and metals to achieve long-term stability.

Recently, mussel-inspired chemistry has shown great potential to build a convenient adhesion of biopolymer to the solid substrate [16,20]. Extensive studies have certified that catechol motifs contribute to strong adhesion [21]. Dopamine (DA), a mussel-inspired polymer with abundant catechol, can form a polydopamine film on different ranges of both inorganic and organic surfaces by spontaneous oxidative polymerization [21,22]. Besides, over the past decade, a variety of catechol-rich chemicals have been connected to the chitosan backbone [23], such as 3,4-dihydroxyhydrocinnamic acid [6,24,25], 3,4-dihydroxybenzealdehyde [26,27], ferulic acid [28,29,30], etc. Gallic acid (GA) is currently the most used phenolic acid to be grafted onto chitosan, owing to its antioxidant properties and low cost [31].

**Scheme 1:**
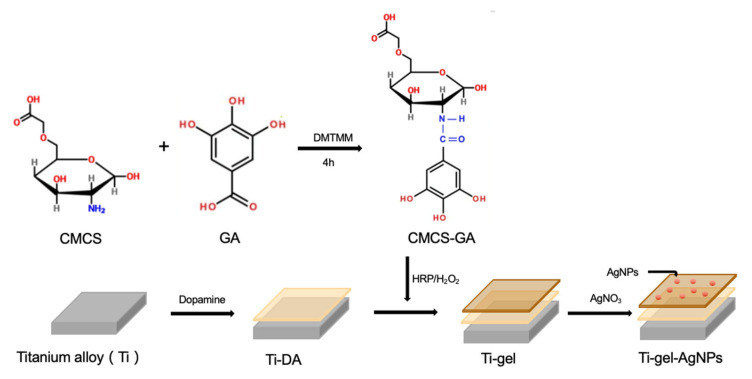
Schematic illustration of the fabrication of the bioactive surface coating onto the titanium alloy surface. Ti-DA represents Ti with dopamine. Ti-gel represents Ti with CMCS-GA-gel. Ti-gel-AgNPs represents CMCS-GA-AgNPs.

In this study, we report a novel bioactive surface coating that consists of a mussel-inspired carboxymethyl chitosan hydrogel loaded with silver nanoparticles (AgNPs) to enhance the bioactive properties of the titanium alloy. As shown in Scheme 1, gallic acid (GA) was successfully grafted onto the carboxymethyl chitosan (CMCS) backbone using DMTMM (4-(4,6-dimethoxy-1,3,5-triazin-2-yl)-4-methylmorpholinium chloride) catalysis. The obtained CMCS-GA can simply form a novel hydrogel coating on the titanium alloy with a dopamine layer, under the catalysis of HRP/H_2_O_2_. It was expected that the resulting hydrogel coating would show strong adhesion to the titanium alloy, and largely enhance the biocompatibility and antibacterial property of titanium alloy. In addition, silver nanoparticles (AgNPs) were synthesized in situ, using the reduction of carboxymethyl chitosan to further enhance the antibacterial properties of the coating. The surface characteristics of CMCS-GA hydrogel coating were tested by Fourier-transform infrared spectroscopy (FTIR), scanning electron microscope (SEM), X-ray photoelectron spectroscopy (XPS), etc. In addition, the antibacterial and cell compatibility of the coating hydrogel were tested in vitro.

## 2. Materials and Methods

### 2.1. Materials

CMCS, GA, 4-(4,6-dimethoxy-1,3,5-triazin-2-yl)-4-methylmorpholinium chloride (DMTMM) were purchased from Macklin Biochemical Co., Ltd. (Shanghai, China). Tris(hydroxymethyl)aminomethane (Tris), AgNO3, and NaOH were purchased from Aladdin Industrial Inc. (Shanghai, China). *Escherichia coli (E. coli, ATCC 25922)* and *Staphylococcus aureus (S. aureus, ATCC 25923)* were purchased from China General Microbiological Culture Collection Center (Beijing, China). All other reagents were of analytical grade. Titanium alloy plates were provided by The General Hospital of the People’s Liberation Army (PLAGH, Beijing, China). Rabbit mesenchymal stem cells (MSCs) were purchased from Procell Life Science & Technology Co., Ltd. (Wuhan, China).

### 2.2. Preparation of CMCS-GA

The grafting of GA onto CMCS was achieved by the reaction between the carboxyl group of GA and the amine group of CMCS catalyzed by DMTMM. Briefly, 0.25 g GA and 0.75 g DMTMM were dissolved in 25 mL deionized water (pH = 7.5) and stirred at room temperature for 20 min under N_2_ to activate the carboxyl group, and this was followed by adding 0.25 g CMCS dissolved in 25 mL deionized water. The reaction was then kept for 4 h at room temperature under N_2_. Then, the product was precipitated with ethanol and filtered with suction to obtain a solid product, and this was followed by lyophilization to obtain the final CMCS-GA. The grafted gallic acid content in CMCS-GA was determined by the Folin–Ciocalteau method (ref). Briefly, 1.0 mL of Folin–Ciocalteau reagent was added to 0.5 mL CMCS-GA solution and reacted for 5 min in the dark. Then, 2.0 mL of sodium carbonate (Na_2_CO_3_) solution (200.0 g/L) was added to the mixture and kept at 30 °C for 1 h. The absorbance was measured at 747 nm by UV-Vis Spectrophotometer (NanoDrop One, Thermo Fisher, Waltham, MA, USA). Gallic acid was used as a standard. The gallic acid content in CMCS-GA was expressed as milligrams of gallic acid equivalent per gram of conjugate (mg GAE/g).

### 2.3. Preparation of CMCS-GA Hydrogel on Titanium Alloy Plate

The clean titanium alloy plate was treated with 5 M NaOH for 6 h at 80 °C. Then, after alkali heat treatment, the titanium alloy plate was soaked in 2% dopamine dissolved in Tris-HCl buffer at pH 8.5 for 24 h (Table 1). The CMCS-GA obtained in the previous step was dissolved in deionized water to make a 5% CMCS-GA solution. Then, 10 μL 3% HRP solution and 10 μL 0.1% H_2_O_2_ solution were mixed with 1 mL CMCS-GA solution, and dropped onto the titanium alloy plate and reacted at 37 °C for 2 h to form the hydrogel. The sample loaded with AgNPs was obtained by immersing the sample in 1 mM AgNO_3_ for 1 h (Table 1).

### 2.4. Fourier-Transform Infrared (FTIR) Spectroscopy

The FTIR spectra of CMCS, GA, and CMCS-GA were measured with the Fourier-transform infrared spectrometer (Vertex 70 V, Bruker, Karlsruhe, Germany) by collecting 32 accumulative scans in 4000–400 cm^−1^ regions, to confirm that gallic acid was successfully grafted onto the chitosan backbone.

### 2.5. Scanning Electron Microscope (SEM)

The titanium alloy plates with different treatments were characterized after the samples were sputtered and plated with gold for about 60 s. The surface morphology of titanium alloy plates was investigated by using a scanning electron microscope (SEM, Thermo Scientific Apreo 2C, Waltham, MA, USA), operating at 5 kV acceleration voltage.

### 2.6. Water Contact Angle

The static water contact angle was measured by the sessile drop method using a contact angle goniometer (JY-82B Kruss DSA, Kruss, Hamberg, Germany). Images of water spreading on the sample surfaces were recorded by a camera, and the contact angle was analyzed with professional software. Three measurements were made for each sample.

### 2.7. X-ray Photoelectron Spectroscopy (XPS)

For chemical composition analysis, specimens were characterized using X-ray photoelectron spectroscopy (Thermo Fisher, ESCALAB 250Xi, Waltham, MA, USA), with a focused monochromatic Mg Kα X-ray source (1253.6 eV) for excitation. The electron take-off angle was 60° in the dry state, and the analyzer was operated in the constant energy mode for all measurements.

### 2.8. Antibacterial Property

The antibacterial property of samples was tested by the zone of inhibition (ZOI) and counting colonies methods. *Escherichia coli* (*E. coli*, *ATCC 25922*) and *Staphylococcus aureus* (*S. aureus*, *ATCC 25923*) were selected as reference strains for antibacterial testing.

For inhibitory zones (ZOI), the nutrient agar medium in the petri dish was inoculated with 100 μL of 10^7^–10^8^ CFU/mL bacteria. Titanium alloy plates were placed on the petri dish and incubated with bacteria at 37 °C for 24 h, and then the diameter of the zone of inhibition was measured. Three replicates were tested for each sample.

For colony count, a single colony was incubated in 4 mL liquid LB medium for 4 h. Then, 100 μL bacterium solution was incubated with 1 g CMCS-GA-gel and CMCS-GA-gel-AgNPs in 4 mL liquid LB medium for 4 h, respectively. After that, the 100 μL 10^5^-fold diluted bacteria solution was spread on the solid medium to count. Three parallels were set for each sample.

### 2.9. Cell Viability

Rabbit mesenchymal stem cells (MSCs) were used to study the adhesion and toxicity of the surface modification of the titanium alloy plate to cells. Titanium alloy plates with different treatments were sterilized in 75% ethanol for 2 h, then washed three times with sterile PBS, and equilibrated in the culture medium for 2 h. MSCs were seeded on the plates with a density of 1×10^5^ cells per well. After 6, 24, and 48 h, the surface of each titanium alloy plate was washed three times with PBS to remove any loosely attached cells. Then, 0.5 mL of 10% CCK-8 medium was added to each well and they continued to incubate for 1 h, then the absorbance was measured at 450 nm (Fluoroskan FL, Thermo Fisher, Waltham, MA, USA). Three parallels were set for each sample. At the same time, fluorescence imaging was used to observe the adhesion of MSCs at different time intervals. The cells were stained with a Live/Dead Cell Double Staining Kit.

## 3. Results and Discussion

### 3.1. FTIR Test

**Figure 1:**
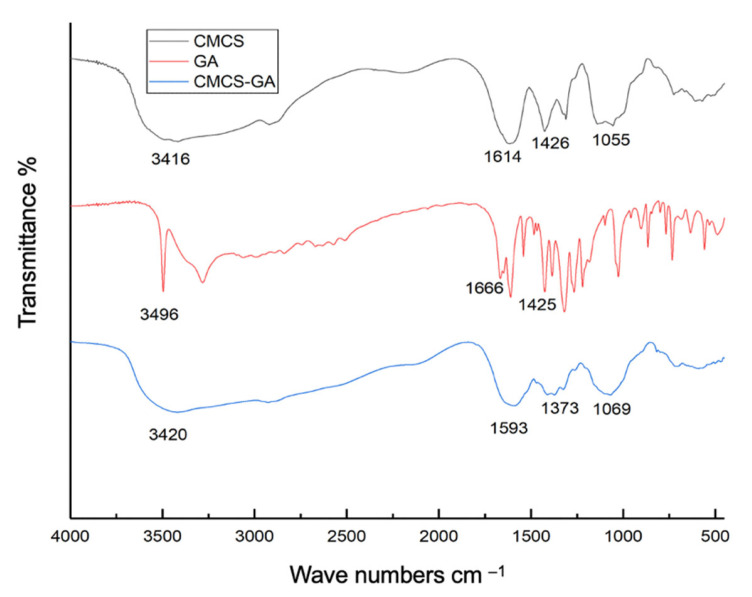
FTIR spectra of CMCS, GA, and CMCS-GA.

To verify whether gallic acid was successfully grafted onto the chitosan backbone, FTIR was used to characterize CMCS, GA, and CMCS-GA. As shown in Figure 1, all samples possess the characteristic peak around 3420 cm^−1^, which is caused by the hydroxyl stretching vibration overlying CMCS and GA. The characteristic peaks at 1614 cm^−1^ of CMCS and 1666 cm^−1^ of GA correspond to the vibration of C=O in the -COOH group. The peaks at 1055 cm^−1^ of CMCS and 1069 cm^−1^ of CMCS-GA are attributed to the C-O-C stretching vibration of the polysaccharide chain. The several characteristic bands of aromatic rings of GA at 1400–1600 cm^−1^ can also be observed. For CMCS-GA, the newly formed bond at 1593 cm^−1^ (C=O,N-H) indicates that amide linkages were established between CS and GA, whereas the peak at 1373 cm^−1^ is designated to O-H of phenolic hydroxyl on GA. All the above results demonstrate the successful conjugation of GA with CMCS. The content of gallic acid in CMCS-GA is determined as 71.7 mg GAE/g.

### 3.2. Surface Morphology

**Figure 2:**
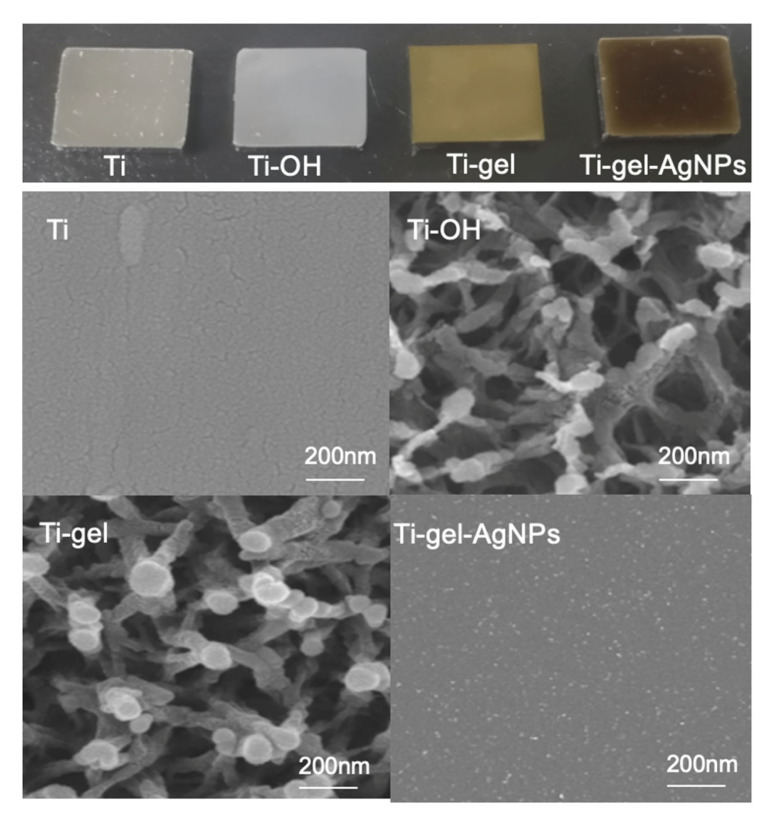
Surface morphology of titanium alloy plates with different treatments.

The surface morphology of the titanium alloy plates was studied during the treatment process. The treatment process involved alkaline heat treatment of the titanium alloy plate to introduce the reactive hydroxyl groups, followed by dopamine treatment to form the polydopamine layer, and the addition of CMCS-GA in order to form a gel. After each treatment step, both a photograph and a SEM image were taken of each sample, for the purpose of comparison. The results show that the untreated titanium alloy plate has a smooth surface and metallic color, which slightly changes to a brighter color, and a uniform porous structure appeared on the surface in the SEM image after the alkaline treatment, as shown in Figure 2. After the dopamine treatment, the formation of the polydopamine layer caused the surface of the titanium alloy to turn brown, as the phenolic hydroxyl group on dopamine is oxidized to a quinone. Furthermore, the application of the prepared CMCS-GA further changed the surface color to a darker brown, and a smooth surface once again replaced the porous structure in SEM image, which can be attributed to the further oxidative cross-linking of phenol in gallic acid and dopamine to form a dense and uniform gel layer on the surface of the titanium alloy.

### 3.3. Water Contact Angle

**Figure 3:**
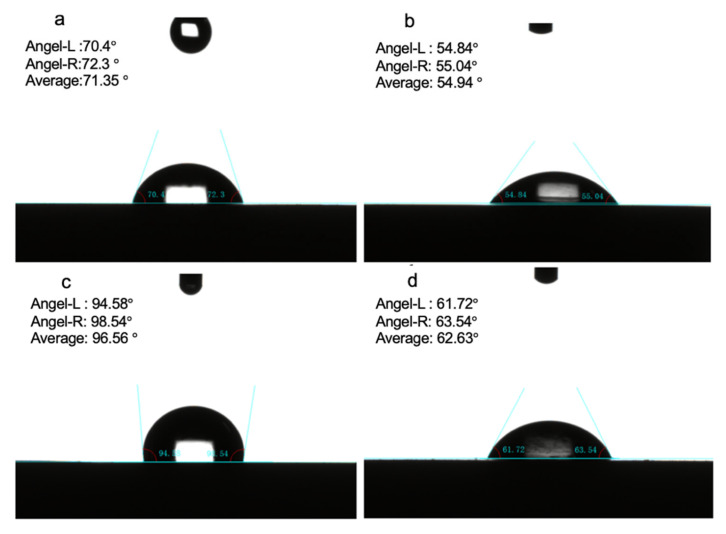
The contact angle image of (**a**) Ti, (**b**) Ti-OH, (**c**) Ti-DA, and (**d**) Ti-gel.

Figure 3 shows the contact angle results of the original titanium alloy (Ti), Ti-OH, Ti-DA, and Ti-gel. For the original titanium, the contact angle is approximately 71° (Figure 3a). After alkali heat treatment, the contact angle decreases to 55° (Figure 3b), which is due to a large number of hydrophilic hydroxyl groups on the surface. The construction of the polydopamine layer destroys the hydrophilic surface formed by alkali heat treatment (Figure 3c). The reason for this is the combination of amine groups and hydroxyl groups during the polymerization of dopamine reduces the number of hydroxyl groups. However, CMCS-GA hydrogel reduces the contact angle of the sample surface to 63° (Figure 3d), as CMCS-GA contains a large amount of hydrophilic carboxyl and hydroxyl groups, making the surface more hydrophilic than the original titanium alloy. The hydrogel improves the wettability of the titanium alloy surface, which can help with the adhesion and proliferation of cells on the surface [32].

### 3.4. Chemical Composition

**Figure 4:**
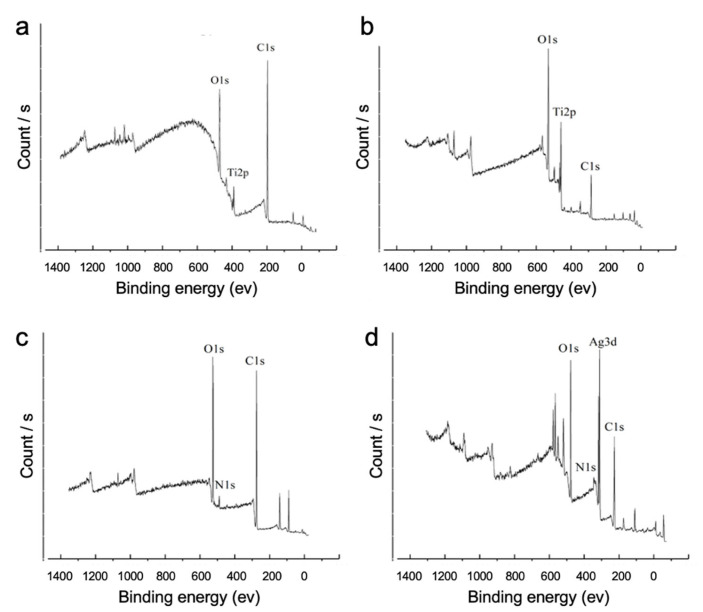
XPS spectra of (**a**) original titanium alloy (Ti), (**b**) Ti-OH, (**c**) Ti-gel, and (**d**) Ti-gel-AgNPs.

The chemical composition of the sample was further tested by XPS. Figure 4 shows the XPS spectra of original titanium alloy (Ti), Ti-OH, Ti-gel, and Ti-gel-AgNPs. In Figure 4a, the peak at bonding energies of 458.1 and 463.8 eV correspond to Ti 2p3/2 and Ti 2p1/2, respectively. The peak of O1s also can be clearly seen at 533.1 eV. This means that the original titanium surface is mainly composed of TiO_2_. Elements C and N are attributed to impurities contained in the titanium alloy and unavoidable ambient air adsorption. After alkali heat treatment (Figure 4b), the peak of O1s still clearly exists in the spectrum, but the peak of C1s is no longer discernible, which proves that the surface of the titanium alloy is covered by many hydroxyl groups. The peak of Ti disappeared in Figure 4c, indicating that the surface of the titanium alloy is covered by a dopamine and gel layer. The peak of Ag3d at 368.4 and 374.4 eV is clearly present in Figure 4d. In relation to Figure 4c, the newly emerged peak results from AgNPs formation.

### 3.5. Antibacterial Activity

It is widely acknowledged that chitosan exhibits a bactericidal effect only at very high concentrations. To improve the antibacterial property of hydrogel, it is worth considering introducing silver nanoparticles, with well-established inhibitory effects. Polysaccharides can be used as reducing agents and stabilizers to provide a green method for simply obtaining silver nanoparticles through in situ reduction [33]. The antibacterial activity test was assessed against *E. coli.* and *S. aureus.*

**Figure 5:**
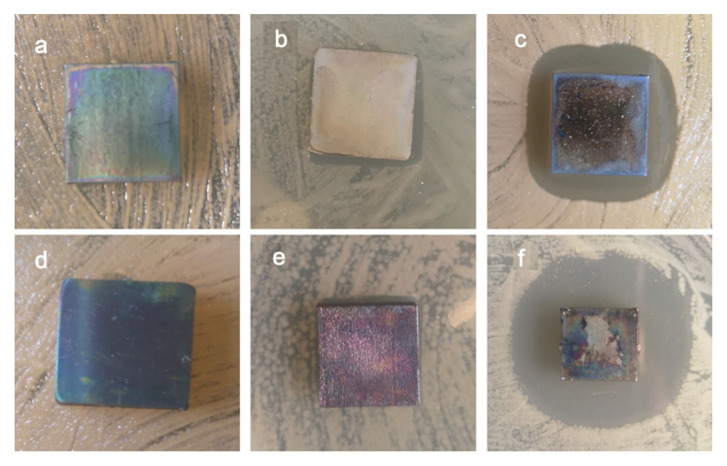
The zone of inhibition (ZOI) of *E. coli.* (**a**–**c**) and *S. aureus.* (**d**–**f**) after incubation with original titanium alloy (**a**,**d**), Ti-gel (**b**,**e**), and Ti-gel-AgNPs (**c**,**f)**.

Figure 5 shows the results of the zone of inhibition (ZOI) test of original titanium alloy (Ti), Ti-gel, and Ti-gel-AgNPs. For bare original titanium, the ZOI is 0 for both *E. coli* and *S. aureus.* After loading the gel layer, the ZOI increases to 1.21 ± 0.05 mm and 1.12 ± 0.03 mm for *E. coli* and *S. aureus,* respectively, which can be attributed to the cationic antibacterial property of CMCS. The zone can be observed more clearly after loading AgNPs; the ZOI is 17 ± 0.20 mm for *E. coli* and 23 ± 0.20 mm for *S. aureus.* The antibacterial activity could be due to the synergistic effects of CMCS and silver particles.

**Figure 6:**
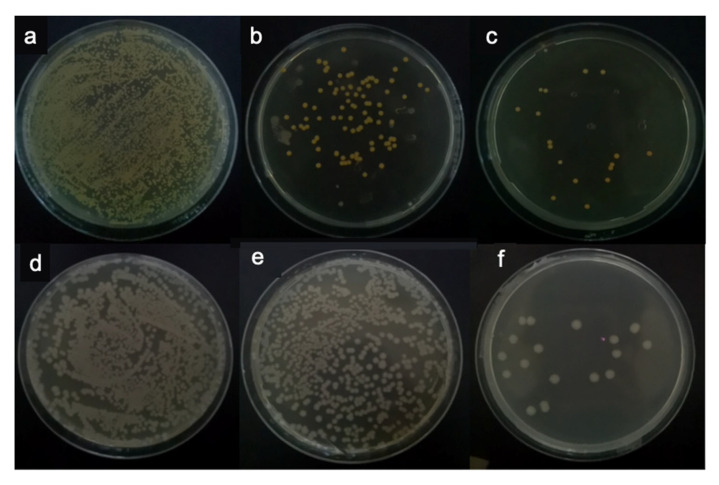
Colonies number of *E. coli.* (**a**–**c**) and *S. aureus.* (**d**–**f**) after incubation with CMCS-GA-gel and CMCS-GA-AgNPs. (**a**,**d**) are blank controls; (**b**,**e**) are the group incubated with CMCS-GA-gel. (**d**,**f**) are the group incubated with CMCS-GA-AgNPs.

The above results were also proven by counting colonies. Figure 6 shows the colonies number of *E. coli* and *S. aureus* after incubation with CMCS-GA-gel and CMCS-GA-AgNPs, respectively. The blank controls of both bacteria contained approximately 10^8^ CFU/plate. The colonies number is reduced to approximately 10^7^ CFU/plate after co-incubation with the CMCS-GA-gel. As a Gram-negative bacterium, *E. coli* contains a lot of hydrophobic lipids and proteins in its cell wall, which makes its cell surface less hydrophilic than Gram-positive bacteria. Therefore, hydrophilic CMCS exhibits a more significant inhibition on *S.aureus.* After loading AgNPs, the colonies number is decreased to 1.7 × 10^6^ CFU/plate for both *S. aureus* and *E. coli.*

### 3.6. Cell Viability

**Figure 7:**
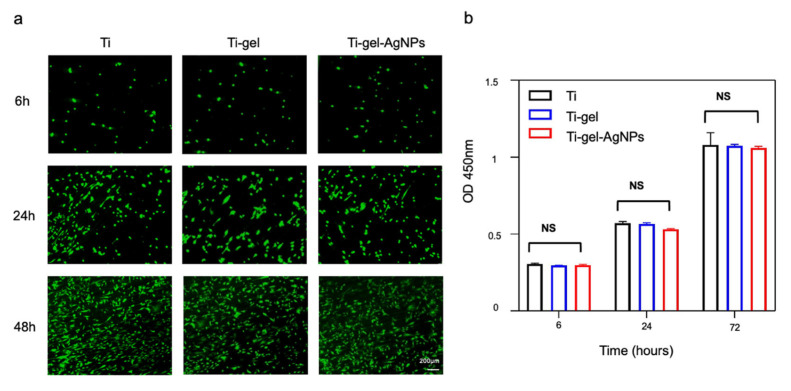
MSCs fluorescence imaging (**a**) and viability (**b**) after co-cultured with Ti, Ti-gel, Ti-gel-AgNPs for 6 h, 24 h, and 72 h. NS means no significant.

The biocompatibility of titanium alloy plates with different treatments was assessed by the in vitro viability of MSCs in direct contact samples, using a CCK-8 assay. The MSCs were seeded on the titanium alloy plates surfaces and cultured for 6 h, 24 h, and 72 h. Cell viability was measured by OD 450 nm with a plate reader. As shown in Figure 7, after 6 h, 24 h, and 72 h incubation, there was no significant difference between the viability of cells incubated in all samples. This result is consistent with that observed by fluorescence imaging, which indicated that MSCs seeded on the hydrogel surfaces were able to proliferate along with increasing culture time. Similar results were reported previously for the biocompatibility of AgNPs-loaded hybrid hydrogels [18,34]. From these results, we suggest that the CMCS-GA-AgNPs composite hydrogels are potential biomaterials with excellent antibacterial activities and without significant cell cytotoxicity.

## 4. Conclusions

In summary, we have demonstrated a novel bioactive surface coating that consists of a mussel-inspired carboxymethyl chitosan hydrogel loaded with AgNPs to enhance the bioactive properties of the titanium alloy. The coating of CMCS-GA-gel could enhance the hydrophilicity of the titanium alloy plate surface, which helps the adhesion and proliferation of cells on the surface. The antibacterial activity test showed that the Ti-gel loaded with AgNPs possessed a significant antibacterial effect. Since *S. aureus* is a Gram-positive bacteria with a more hydrophilic surface, CMCS-GA-gel is more efficient against *S. aureus* than *E. coli.* Ti-gel and Ti-gel-AgNPs show similar biocompatibility in terms of MSCs adhesion and proliferation, in comparison with Ti. These results suggest that this CMCS-GA-gel provides a promising alternative as a design for a multifunctional hydrogel coating for Ti implants that supports cell adhesion, spreading, and drug loading.

## Figures and Tables

**Table 1:** Titanium alloy plates with different treatments.

	Alkali Heat Treatment	Dopamine	CMCS-GA-gel	AgNO_3_
Ti	✗	✗	✗	✗
Ti-OH	✓	✗	✗	✗
Ti-DA	✓	✓	✗	✗
Ti-gel	✓	✓	✓	✗
Ti-gel-AgNPs	✓	✓	✓	✓

## Data Availability

The original contributions presented in the study are included in the article, further inquiries can be directed to the corresponding authors.
